# miR-199a-5p Reduces Chondrocyte Hypertrophy and Attenuates Osteoarthritis Progression via the Indian Hedgehog Signal Pathway

**DOI:** 10.3390/jcm12041313

**Published:** 2023-02-07

**Authors:** Lei Huang, Meng Jin, Ruiying Gu, Kunlin Xiao, Mengnan Lu, Xinyu Huo, Mengyao Sun, Zhi Yang, Zhiyuan Wang, Weijie Zhang, Liqiang Zhi, Ziang Meng, Jie Ma, Jianbing Ma, Rui Zhang

**Affiliations:** 1 Department of Joint Surgery, Honghui Hospital, Xi’an Jiaotong University, Xi’an 710054, China; 2 Translational Medicine Center, Honghui Hospital, Xi’an Jiaotong University, Xi’an 710054, China; 3 School of Basic Medical Science, Xi’an Jiaotong University Health Science Center, Xi’an 710049, China; 4 Department of Mathematics and Computing Science, Simon Fraser University, Vancouver, BC V6B 5K3, Canada

**Keywords:** miR-199a-5p, osteoarthritis, cartilage, chondrocyte, intra-articular injection

## Abstract

Osteoarthritis (OA), the most common type of arthritis, is an age-associated disease, characterized by the progressive degradation of articular cartilage, synovial inflammation, and degeneration of subchondral bone. Chondrocyte proliferation is regulated by the Indian hedgehog (IHH in humans, Ihh in animals) signaling molecule, which regulates hypertrophy and endochondral ossification in the development of the skeletal system. microRNAs (miRNAs, miRs) are a family of about 22-nucleotide endogenous non-coding RNAs, which negatively regulate gene expression. In this study, the expression level of IHH was upregulated in the damaged articular cartilage tissues among OA patients and OA cell cultures, while that of miR-199a-5p was the opposite. Further investigations demonstrated that miR-199a-5p could directly regulate IHH expression and reduce chondrocyte hypertrophy and matrix degradation via the IHH signal pathway in the primary human chondrocytes. The intra-articular injection of synthetic miR-199a-5p agomir attenuated OA symptoms in rats, including the alleviation of articular cartilage destruction, subchondral bone degradation, and synovial inflammation. The miR-199a-5p agomir could also inhibit the Ihh signaling pathway in vivo. This study might help in understanding the role of miR-199a-5p in the pathophysiology and molecular mechanisms of OA and indicate a potential novel therapeutic strategy for OA patients.

## 1. Introduction

Osteoarthritis (OA), the most common type of arthritis, is an age-associated disease, characterized by the progressive degradation of articular cartilage, synovial inflammation, and degeneration of subchondral bone [1,2]. Its progression is affected by multiple factors, including hereditary contributions, mechanical factors, trauma, and aging [3]. According to twin studies, genetic factors might account for 70% of the OA affecting specific joints [4].

Chondrocyte proliferation is regulated by the Indian hedgehog (IHH in humans, Ihh in animals) signal molecule, which is a part of the hedgehog family and regulates hypertrophy and endochondral ossification in the development of the skeletal system [5,6,7]. Lin et al. reported that the higher levels of Ihh in chondrocytes could induce more severe OA-like pathologies, such as chondrocyte hypertrophy and cartilage degradation in the postnatal cartilage in genetically engineered mice [8]. Zhou et al. observed mild OA features in mice with the conditional deletion of Ihh combined with the OA-induced surgery of partial medial meniscectomy (PMM). On the other hand, the control mice with PMM surgery showed significantly more severe damage to the cartilage, thereby demonstrating the protective role of Ihh deletion in these mice [9]. Wei et al. reported an increase in the Ihh expression level in the OA cartilage and synovial fluid as compared to the normal control samples. The overexpression of IHH significantly promoted the expression level of matrix metallopeptidase 13 (MMP13) in human chondrocytes, while its knockdown showed opposite effects [10]. Therefore, inhibiting IHH signaling might be a possible strategy to prevent the progression of OA.

microRNAs (miRNAs, miRs) are a family of about 22-nucleotide endogenous small non-coding RNAs, which negatively regulate gene expression by cleaving the mRNAs or at post-transcriptional levels [11]. They recognize the targeted genes and regulate their expression by binding to the 3′-UTR of their target mRNAs [12]. A large number of miRNAs are involved in the anabolism and catabolism of cartilage, as well as in the pathogenesis of OA. Among the OA-related miRNAs, miR-140 has been extensively studied [13]. The loss of miR-140 could develop OA phenotypes in the mice, while its overexpression in cartilage showed resistance to the antigen-induced arthritis [14]. The intra-articular injection of miR-140 could modulate the extracellular matrix homeostasis and attenuate the OA progression in rats [15]. 

The plasma expression levels of miR-199a-5p were lower in late OA patients than those with OA at the earlier stages [16]. miR-199a-5p has been reported to be strongly upregulated during chondrogenesis from murine mesenchymal stem cells in response to stimulation by TGFβ3 [17]. The miR-199a-5p overexpression could reverse the visfatin-induced elevation of two proinflammatory cytokines, IL-6 and TNFα, in the human synovial fibroblasts [18]. Proinflammatory cytokines, such as IL-1β, TNFα, and IL-6 might cause synovial inflammation and cartilage destruction during the progression of OA. Indian hedgehog signal has been found to be regulated by proinflammatory factor IL-1β in bovine articular chondrocytes [19]. The current study found that miR-199a-5p could directly target IHH in primary human chondrocytes (PHCs) and attenuate OA progression in rats. 

## 2. Materials and Methods

### 2.1. Collection of Human Articular Cartilage and Preparation of Paraffin Section 

The study was approved by the Ethical Committee of Xi’an Honghui Hospital, Xi’an Jiaotong University, China (No.201902062). The OA patients were diagnosed based on the modified Outerbridge classification by Xi’an Honghui Hospital. All the articular cartilage tissues affected by OA were obtained from patients undergoing the surgery of knee arthroplasty. The initial cartilage specimens of patients and healthy controls were collected from the previous study [20]. The later articular cartilage tissues were collected from 16 patients. Their detailed information is listed in Table S1 (Supplementary Materials, Table S1). All the participants provided informed consent to participate in the study. The articular cartilage tissues were fixed, decalcified, embedded in paraffin, and finally cut into 5-μm thick sections. 

### 2.2. Bioinformatics Analysis of the Gene Expression Profiles in Rats

The dataset GSE30322 for gene expression profiles was downloaded from the Gene Expression Omnibus (GEO) database (https://www.ncbi.nlm.nih.gov/geo/, accessed on 30 July 2021). A total of ninety adult male Sprague-Dawley (SD) rats were equally divided into two groups: the experimental group (E Group) and the sham group (S Group). The subchondral bones of the two groups were collected after 1 week of surgery. Further information can be found in the previously published study [21]. 

R package v. 3.6.3 was used to identify the differentially expressed genes (DEGs) between the two groups. The statistically significant difference was identified at Padj < 0.01 and |Log2FC| > 1 using the R package Deseq2 [22]. A volcano plot was constructed using Enhanced Volcano (v. 1.7.16) and ggplot2 R packages [23]. The Gene Ontology (GO) and Kyoto Encyclopedia of Genes and Genomes (KEGG) enrichment analyses were performed using the “org.Hs.eg.db” (v. 3.11.4) and “clusterProfiler” (v. 3.17.3) in the R package.

### 2.3. Immunohistochemistry (IHC) 

The cartilage sections were deparaffinized, rehydrated, antigen repaired, and were incubated with an IHH antibody at the dilution of 1:50 (Abcam) overnight. The primary antibodies for the protein detection in rat tissue sections included anti-IHH (1:200; Abcam), anti-Gli1 (1:200; Abways), anti-Runx2 (1:200; CST), anti-Mmp13 (1:50; Boiss) and anti-Col2a1 (1:50; Boster). The protein samples were stained using the Diaminobenzidine (DAB) substrate kit and counterstained with hematoxylin (Boster). Image J software was used to analyze the mean optical density of IHH staining.

### 2.4. Fluorescence In Situ Hybridization (FISH) 

A Cy3-labeled miR-199a-5p probe (5′-CCAATCTCTTCCCACTTGACAGGTGCC-3′) was designed and synthesized by GenePharm. The FISH Kit of GenePharm was used for the detection of probe signals following the manufacturer’s instructions. The images were captured using Zeiss LSM800 Laser Scanning Confocal Microscope. 

### 2.5. Cell Culture 

The smooth layers of the patients’ articular cartilage tissues were carefully isolated and cut into pieces. Then, the pieces were digested with 0.25% trypsin (Hyclone) for 20 min and 0.2% type II collagenase (Sigma) at 37 °C for 3 h. The articular chondrocytes were washed twice with PBS and centrifuged at 1000 g for 10 min. Finally, the cells were seeded into a 6 cm dish with F12/DMEM (HyClone), containing 10% FBS (Gibco) and 1% penicillin/streptomycin (Thermo, Waltham, MA, USA). The 293T cells were cultured in high-glucose DMEM (HyClone) supplemented with 10% FBS and 1% penicillin/streptomycin. 

### 2.6. OA Cell Model Induction, RNA Extraction, and Quantification Analysis

IL-1β was used for the induction of the OA cell model in the primary chondrocytes isolated from the normal cartilage tissues in patients with IL-1β [24,25]. Briefly, the PHCs were seeded into a 6-well plate at a cell density of 2 × 104 cells/cm2 and induced with 10 ng/mL IL-1β (Sigma) for 24 h overnight. Total RNA was extracted using Trizol reagent (Thermo) following the manufacturer’s instructions. The cDNA for mRNA quantitation was synthesized with a 2 μg RNA using the RevertaidTM First Strand cDNA Synthesis kit (Fermentas). Meanwhile, the cDNA for miRNA quantitation was synthesized with 1 μg RNA using a universal miRNA one-step RT kit (Takara Bio Inc, Kusatsu, Shiga). The gene expression levels were detected with quantitative PCR (qPCR) using the SYBR Green System (Roche). The expression levels of miRNAs were detected using a universal miRNA one-step RT kit and normalized with the U6. GAPDH was used for the normalization of mRNA expression levels. The primers for qPCR were listed in Table S2 (Supplementary Materials, Table S2). 

### 2.7. Construction of Recombinant DNA and Sequence Mutagenesis

A 500-bp 3′-UTR fragment within the IHH gene containing the predicted miR-199a-5p binding sites (5′-ACACTGG-3′) was synthesized by GenePharm. The synthesized sequence was cloned into a pmirGLO luciferase vector (Promega, Madison, WI, USA) between the SacI and XbaI restriction sites. The DNA sequences were found to be consistent with the RCh38/hg38 reference sequence from the UCSC genome browser named a wild-type (WT) vector. The mutated sequences containing all seven nucleotides in the predicted seed-pair region (5′-TGTGACC-3′) were synthesized and cloned into the pmirGLO vector named a mutant-type (MUT) vector. Both the vectors were validated using restriction digestion and Sanger sequencing.

### 2.8. Transfection

The miR-199a-5p sequence (miR-199a-5p mimics) and negative control (mimic NC) were synthesized by GenePharm. The sequences are listed in Table S3 (Supplementary Materials, Table S3). HEK293T cells were seeded into a 48-well plate at density of 3 × 10^4^ cells/well. After 24 h, the HEK293T cells were co-transfected with 50 nM mimic NC or miR-199a-5p mimics with 10 ng WT or MUT vector using Lipofectamine 3000 (Thermo). The cells were then harvested after 24 or 48 h for the dual-luciferase reporter gene assay. The PHCs were seeded into six-well plates at density of 2 × 10^5^ cells/well. After 24 h, 50 nM miR-199a-5p mimics or NC were transfected into PHCs for the next experimental steps.

### 2.9. Dual-Luciferase Reporter Gene Assay

The activity of the luciferase reporter gene was determined using a Dual-Luciferase Reporter Assay System (Promega) following the manufacturer’s instructions. The results were obtained using a plate-reading luminometer. Each sample was tested in quadruplicate and the activity of luciferase was calculated against a blank control. The relative activity was calculated as the ratio of firefly to renilla luciferase activities.

### 2.10. Ad-IHH Construction and Infection

The human IHH gene sequence was amplified using PCR and cloned into the pAdEasy-EF1-MCS-CMV-GFP adenoviruses vector (Hanbio Tech, Shanghai, China). The primer sequences were shown in Table S2 (Supplementary Materials, Table S2). After the transfection of miRNA mimic NC or miR-199a mimics into PHCs, the adenoviruses IHH (ad-IHH) and adenoviruses NC (ad-NC) were also transfected into PHCs respectively. The PHCs were harvested after 48 h for western blot analysis. 

### 2.11. Protein Extraction and Western Blot Analysis

The PHCs were harvested and lysed using RIPA buffer (Beyotime) supplemented with protease inhibitor (Roche). The cell lysate was centrifuged at 12,000 g and 4 °C for 15 min and the protein concentrations were measured using BCA Protein Assay Kit (Thermo). A total of 20 μg of proteins from each sample were separated on 8–10% SDS/PAGE. The primary antibodies included IHH (1:1000; Abcam), ACAN, GLI1, COL10 (1:400; Abways), RUNX2 (1:1000; CST), COL2A1 (1:200; Boster), ADAMTS5, MMP13 (1:500; Bioss), and GAPDH (1:5000; Abways). The detailed information of all antibodies was shown in Table S4. ImageJ software was used to quantify the relative expression levels of the proteins. 

### 2.12. Induction of Rat Knee OA Model and Intra-Articular Injection

The rat knee OA model was established by anterior cruciate ligament transection (ACLT) with complete medial meniscectomy (MMx) [26]. A total of 18 10-week-old male Sprague-Dawley (SD) rats were used in this study, 18 of which were subjected to the ACLT + MMx surgery on their right legs. All rats were supplied with food and water ad libitum. After 2 weeks, the model rats were randomly divided into two groups, which were intra-articularly injected with 5 nmol agomir NC or 5 nmol agomir miR-199a-5p dissolved in 50 μL of saline. Meanwhile, the sham group rats were intra-articularly injected with 50 μL saline. After 6 weeks, the right knee samples were harvested and embedded in paraffin. 

### 2.13. Histological Staining and Assessment 

The paraffin blocks were sagittally sectioned through the medial femoral condyle and the sections were then stained with hematoxylin and eosin (HE), toluidine blue (TB), and Safranin O and fast green (SOFG) solution (Solarbio, Shanghai, China) respectively. The level of cartilage deterioration and severity of knee OA were determined using the OA Research Society International (OARSI) scoring system containing six OA grades and four OA stages and ranging from 0 to 24, and the modified Mankin criteria ranging from 0 to 14 [27,28]. Synovitis was assessed by HE staining using a modified scoring system, which focused on the pannus formation (score range 0–3), synovial membrane thickening (score range 0–3), and sub-synovial inflammation (score range 0–3) [29]. The bone volume to tissue volume (BV/TV) ratio, measured using microCT, was used as an index to assess the quality of subchondral bone, while the bone area to tissue area (Ba/Ta) ratio, calculated on two-dimensional slides in histomorphometry, was used as reference [30]. Image J software was used to calculate the ratio of trabecular bone area and tissue area. All the evaluations were independently performed by two researchers. 

### 2.14. Statistical Analyses

All data were presented as the mean ± standard error of the mean (SEM). Student’s *t*-test was used to evaluate the independent data which conform to normal distribution and homogeneous variance between two experimental groups. One-way analysis of variance (ANOVA) was performed among multiple groups and Tukey correction was used for pairwise comparisons. Two-way ANOVA was performed to test the data of the dual-luciferase reporter gene assay and Sidak correction was used for pairwise comparisons. All the statistical analyses were performed using SPSS18.0 software. *p* < 0.05 was considered as statistically significant. 

## 3. Results

### 3.1. IHH Expression Was Increased in Damaged and Deep Zone Articular Cartilage in Human Samples

**Figure 1:**
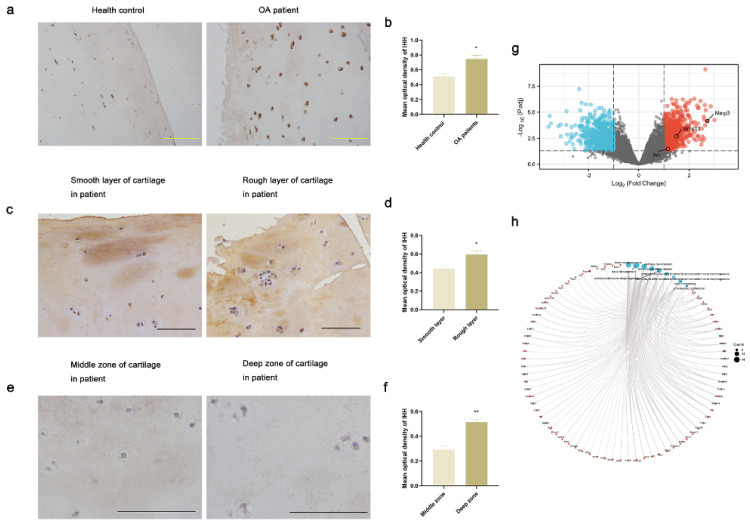
IHH expression was increased in the damaged and deep zone cartilage in human samples and in the subchondral bone in OA rats. (**a**–**f**) Immunohistochemical staining images and quantification analysis of IHH in the articular cartilage of healthy controls compared with those of OA patients, in the smooth layer compared with the rough layer of articular cartilage in OA patients, and in the middle zone compared with the deep zone of articular cartilage in OA patients. (* *p* < 0.05, ** *p* < 0.01; *n* = 3 for each group). (**g**) DEGs in the subchondral bone between sham and OA groups in rats. The expression of Ihh, Mmp13 and Mmp13 was increased in OA rats. (**h**) KEGG and GO enrichment analysis of DEGs. Blue points represent KEGG pathways and GO terms associated with cartilage and bone development, and red points represent related genes. The detailed information of enrichment analysis was shown in Table S5. Scale bar represents 100 μm.

The super layer of articular cartilage was undamaged and smooth in the healthy controls, while it was damaged and appeared rough in the OA patients. The smooth and rough cartilage layers coexisted in the same patient, reflecting the cartilage being undamaged and damaged respectively [25]. The results of IHC in the articular cartilage of OA patients and healthy individuals revealed that the IHH expression level was significantly higher in damaged cartilage compared with undamaged cartilage (Figure 1a–d). Moreover, the IHH expression level was significantly elevated in the deep zone cartilage as compared to that in the middle zone cartilage (Figure 1e,f). In the deep zone articular cartilage tissues, most of the chondrocytes were in the state of terminated hypertrophy [31].

### 3.2. Ihh Expression Was Increased in Subchondral Bone in OA Rats

A total of 15,733 DEGs were identified in the subchondral bone between the S and E groups including 713 upregulated genes and 698 downregulated genes. The Mmp3, Mmp13, and Ihh genes were upregulated in the OA rats as shown in the volcano plot (Figure 1g). The enrichment analysis of the DEGs suggested that they were involved in different roles in bone and chondrocyte (Figure 1h).

### 3.3. miR-199a-5p Expression Was Decreased in OA PHCs and Damaged Cartilage 

**Figure 2:**
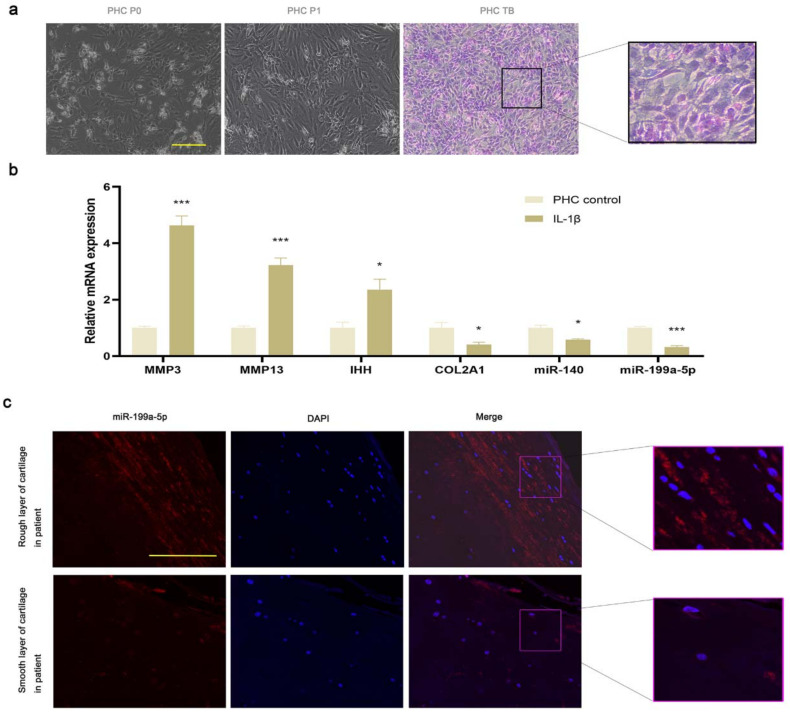
The expression of miR-199a-5p was reduced in OA PHCs and damaged cartilage tissues. (**a**) Light microscopy images of passage 0 and passage 1 PHCs, and toluidine blue staining for the passage 1 PHCs. (**b**) Relative mRNA and miRNA expression levels in chondrocytes. (* *p* < 0.05, *** *p* < 0.001; *n* =3 for each group). (**c**) FISH of miR-199a-5p in the smooth layer of articular cartilage tissues compared with the rough layer of articular cartilage tissues in OA patients. Scale bar represents 100 μm.

The human articular chondrocytes were spindle-shaped and showed positive results in the toluidine blue staining (Figure 2a). The IL-1β-induced PHCs showed a significant increase in the expression levels of MMP3 and MMP13 and a significant decrease in the COL2A1 level as compared to the control group, suggesting the successful induction of the OA model. The expression level of OA-associated miR-140 was lower in the IL-1β-induced PHCs as compared to the control group; these results were consistent with the previous study [32]. The IHH expression level was significantly higher in the OA cells as compared to the control group, while that of miR-199a-5p was significantly lower (Figure 2b). In the damaged cartilage tissues as compared to the undamaged cartilage tissues, the expression level of miR-199a-5p detected using FISH was lower (Figure 2c). 

### 3.4. miR-199a-5p Directly Regulated IHH in PHCs

**Figure 3:**
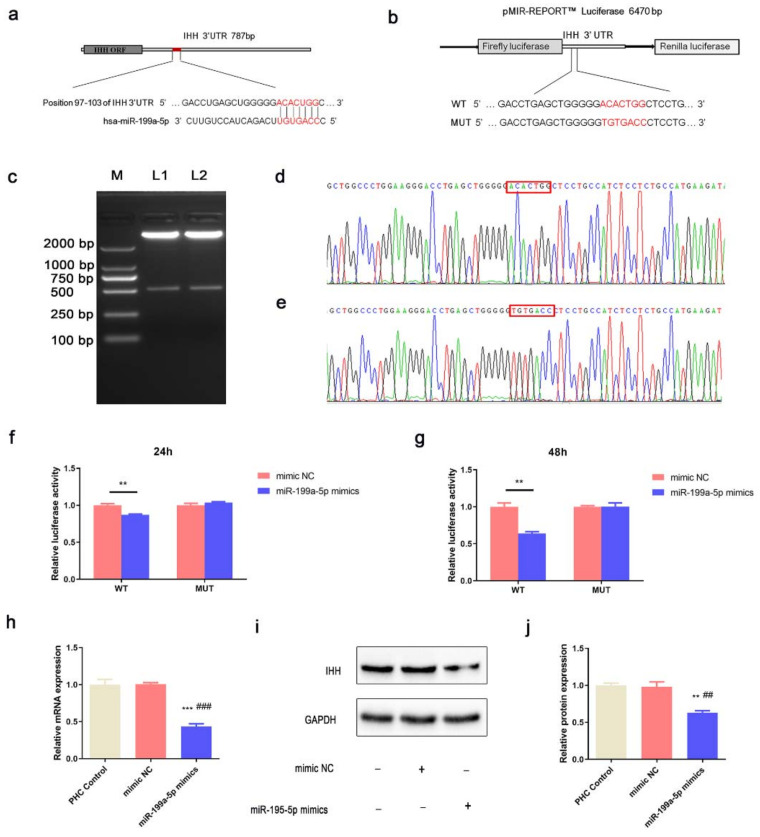
miR-199a-5p directly regulated the IHH expression in PHCs. (**a**) Schematic diagram of the base pairing between miR-199a-5p and IHH 3′ UTR. The putative seed-pair region is shown in red. (**b**) Schematic diagram of the WT and MUT constructs. The putative seed sequence and point mutations in the constructs are shown in red. (**c**) Results of the restriction digestion. M represents the DL2000 marker, lane 1 and lane 2 show the restriction digestion results of WT and MUT constructs respectively. (**d**,**e**) Partial sequencing results of WT and MUT constructs. Nucleotides in the red boxes represent the binding site in IHH 3′ UTR. (**f**,**g**) Dual-luciferase reporter assay of mimic NC and miR-199a-5p mimics co-transfected with WT and MUT constructs into the HEK293T after 24 h and 48 h. (**h**) Relative mRNA expression of the IHH in PHCs after 24 h of transfection. (**i**) Western blot of IHH in PHCs after 48 h of transfection. (**j**) Statistical analysis of the western blot results from 3 independent experiments. (** *p* < 0.01, *** *p* < 0.001 versus mimic NC; ## *p* < 0.01, ### *p* < 0.001 versus miR-199a-5p mimic group; *n* = 3 for each group).

IHH was predicted to be targeted by miR-199a-5p by the TargetScan online website (https://www.targetscan.org/vert_80/, accessed on 3 September 2018) (Figure 3a). The binding sites in the MUT recombinant vector were complementary to those in the WT recombinant vector (Figure 3b). In the restriction digestion products, a 7350-bp band indicated the pmirGLO vector, while the 500-bp band represented the IHH 3′ UTR (Figure 3c). The mutations in the binding sites of the MUT recombinant vector were generated based on the A-T and C-G substitution criteria (Figure 3d,e).

In the HEK293T cells co-transfected with WT vector, the luciferase activity decreased significantly compared to the miR-199a-5p mimic group and compared to the mimic NC group after 24 h and 48 h. Nevertheless, the luciferase activity in cells co-transfected with MUT vector showed no significant difference in the miR-199a-5p mimic and mimic NC group, suggesting that miR-199a-5p could directly bind to the IHH 3′ UTR and suppress its expression (Figure 3f,g). In PHCs, the mRNA and protein levels of IHH were found to be lower in the miR-199a-5p mimic group as compared to those in the control and mimic NC groups (Figure 3h–j). These results suggested that miR-199a-5p downregulated the endogenous expression of IHH in PHCs.

### 3.5. miR-199a-5p Regulated IHH Signal Pathway to Inhibit Chondrocyte Hypertrophy and Matrix Degradation in PHCs

**Figure 4:**
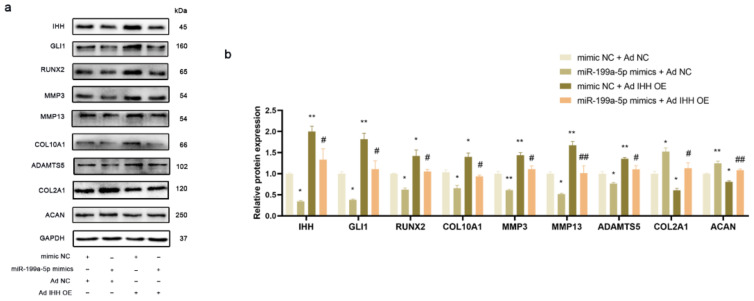
miR-199a-5p regulated IHH signal pathway and matrix degradation in PHCs. (**a**) Western blot analysis of IHH, GLI1, GLI2, RUNX2, COL10A1, MMP13, ADAMTS5, COL2A1, and ACAN normalized with GAPDH in the PHCs. (**b**) Statistical analysis of the western blot results from 3 independent experiments. (* *p* < 0.05, ** *p* < 0.01 versus mimic NC + ad NC group; # *p* < 0.05, ## *p* < 0.01 versus mimic NC + ad IHH OE group; *n* = 3 for each group).

The ad-IHH was transfected into the PHCs for upregulating the IHH expression. As shown in Figure 4a,b, the co-transfection of ad-NC and miR-199a-5p mimics significantly decreased the IHH expression level as compared to that in the cells co-transfected with ad-NC and mimic NC. This decrease in the IHH expression was restored by the overexpression of IHH and miR-199a-5p reversed the overexpression. Meanwhile, miR-199a-5p could significantly decrease the GLI1 expression and the decrease of GLI1 expression was restored by IHH overexpression, suggesting that miR-199a-5p could inhibit the GLI1 expression by regulating the IHH. Gli1 could activate the Runx2 expression and promote chondrocyte hypertrophy [33]. In this study, the protein expression trend of RUNX2 and COL10A1, associated with the chondrocyte hypertrophy, was consistent with that of the IHH and GLI1 in PHCs, suggesting that miR-199a-5p could regulate the IHH signal pathway and chondrocyte hypertrophy. Furthermore, miR-199a-5p decreased the expression levels of catabolic extracellular matrix proteins, including MMP3, MMP13, and ADAMTS5, which were then restored by the IHH overexpression, while the expression levels of anabolic extracellular matrix proteins, including COL2A1 and ACAN showed opposite effects.

### 3.6. Intra-Articular Injection of miR-199a-5p Attenuated OA Progression

**Figure 5:**
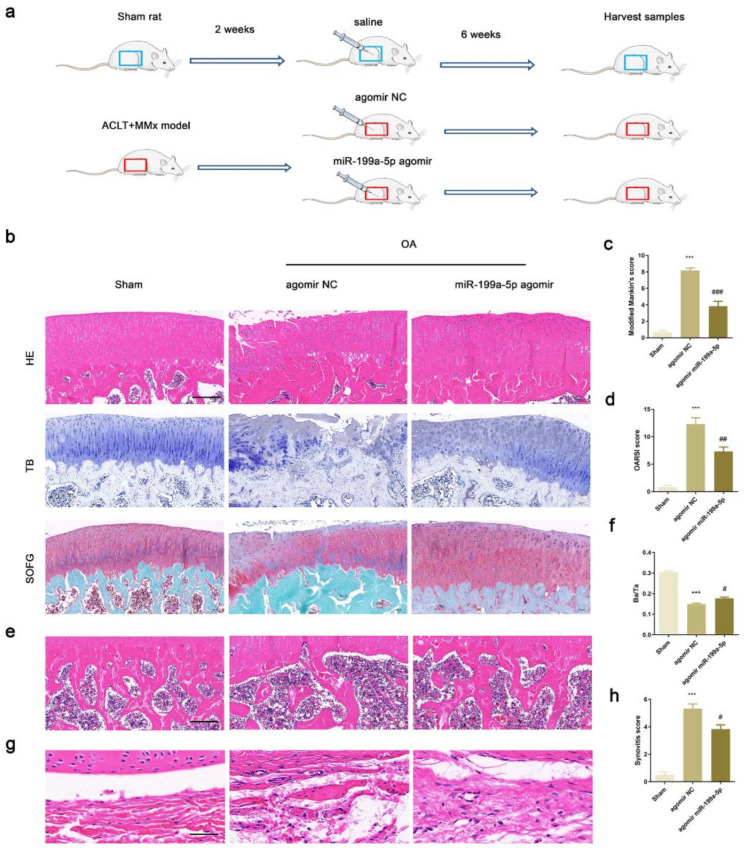
Intra-articular injection of miR-199a-5p attenuated OA progression in rats. (**a**) Flowchart of the experiment. (**b**) HE, TB, and SOFG staining of the articular cartilage tissues in rats. (**c**,**d**) Modified Mankin’s score and OARSI analysis in rats. (**e**) HE staining of subchondral bone. (**f**) The statistical ratio of bone area to the tissue area in the OA rats. (**g**) HE staining of the synovium. (**h**) Synovitis score in rats. Scale bar represents 200 μm. HE, hematoxylin, and eosin; TB, toluidine blue; SOFG, Safranin O and fast green. Scale bar represents 100 μm. (*** *p* < 0.001 versus sham group; # *p* < 0.05, ## *p* < 0.01, ### *p* < 0.001 versus agomir NC group; *n* = 6 for each group).

The knee joints of OA rats including articular cartilage, subchondral bone, and synovium were harvested after 8 weeks of surgery. The histological staining results showed that the articular cartilage was damaged with decreased thickness in the OA rats intra-articularly injected with agomir NC as compared to the sham group rats. In the OA rats injected with miR-199a-5p agomir as compared to those injected with agomir NC, the damaged cartilage was partially repaired (Figure 5b). The modified Mankin’s and OARSI scores decreased significantly in the miR-199a-5p agomir group compared to the agomir NC group (Figure 5c,d). The staining and statistical analysis of subchondral bone area demonstrated that the trabecular bone area significantly increased in the miR-199a-5p agomir group as compared to the agomir NC group (Figure 5e,f). The synovial inflammation was alleviated in the miR-199a-5p agomir group as compared to the agomir NC group (Figure 5g,h). These results suggested that miR-199a-5p could attenuate the OA progression in vivo.

### 3.7. Intra-Articular Injection of miR-199a-5p Regulated Ihh Signal Pathway and Inhibited Matrix Degradation

**Figure 6:**
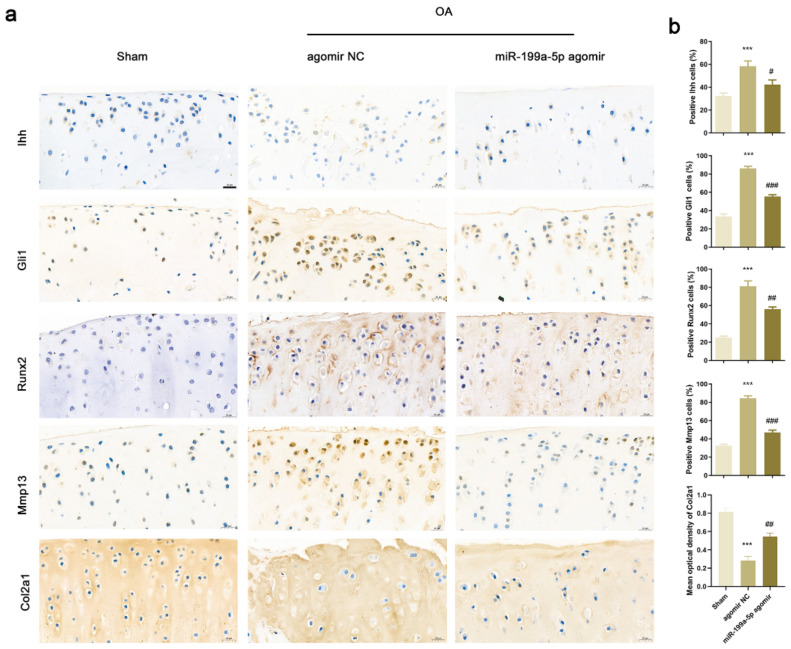
miR-199a-5p regulated molecular markers associated with chondrocyte hypertrophy and matrix degradation via Ihh signaling pathway in rats. (**a**) Immunohistochemical staining of Ihh, Gli1, Runx2, Mmp13, and Col2a1. (**b**) Statistical analysis of the chondrocytes positive for Ihh, Gli1, Runx2, Mmp13, and Col2a1. Scale bar represents 20 μm. (*** *p* < 0.001 versus sham group; # *p* < 0.05, ## *p* < 0.01, ### *p* < 0.001 versus agomir NC group; *n* = 6 for each group).

Immunohistochemical staining was performed to identify the expression levels of genes associated with the Ihh pathway and matrix degradation (Figure 6a). The protein levels of Ihh, Gli1, Runx2, and Mmp13 were higher in the OA rats of the agomir NC group as compared to those in the sham group rats. Similarly, the percentage of Ihh, Gli1, Runx2, and Mmp13-positive articular chondrocytes significantly increased in the OA rats (Figure 6b). Conversely, the expression trend and mean optical density of Col2a1 were significantly lower in the OA rats of the agomir NC group as compared to those in the sham group. The expression and proportion of the positive cells in articular cartilage tissues of OA rats intra-articularly injected with miR-199a-5p agomir were reversed as compared to those injected with agomir NC, suggesting that miR-199a-5p could inhibit matrix degradation by regulating the Ihh signal pathway.

## 4. Discussion

The current study showed that IHH was correlated with cartilage degradation and chondrocyte hypertrophy. The expression of IHH was increased, while that of miR-199a-5p was decreased in damaged cartilage and OA cell model. miR-199a-5p could target IHH and regulate the IHH signal pathway in PHCs, and alleviate OA progress in rats. The bioinformatics analysis of the dataset demonstrated that Ihh expression increased in the subchondral bone in the early OA rats. At the early OA stage, the cartilage tissue was still intact and the upregulated Ihh in the subchondral bone could trigger hypertrophy signals in the chondrocytes. The chondrocyte hypertrophy caused the articular cartilage degeneration, which occurred in late OA both in animals and humans [21]. The proper regulation of chondrocyte hypertrophy is essential in postnatal cartilage homeostasis and Ihh overexpression might lead to the accelerated chondrocyte hypertrophy, resulting in reduction of joint cartilage [7]. 

In vertebrates, Ihh could bind to its receptor to active Smo, and the activated Ihh signal was mediated by the Gli transcription factor family (Gli1, Gli2, and Gli3). Gli1 is a strong transcriptional activator and one of the target genes of Ihh signaling [34]. Gli1/2 could increase the Col10a1 promoter activity and the response element was found in its basic promoter. In addition, the Gli1/2-linked Runx2 could bind to the endogenous Col10a1 promoter in chondrocytes [35]. The transcription factor Runx2 could regulate the Col10a1 expression and matrix mineralization of chondrocytes [36]. Therefore, it was suggested that Gli1/2 and Runx2 might synergistically induce the Col10a1 expression to stimulate the chondrocyte hypertrophy and induce the matrix degradation. The current study showed that IHH overexpression could significantly increase the expression level of GLI1 transcription factor as well as the downstream factors of hypertrophy markers, including RUNX2 and COL10A1, which consequently led to matrix degradation in the PHCs. The expression levels of MMP13 and ADAMTS5 were upregulated, while those of COL2A1 and ACAN were downregulated, indicating that extracellular matrix metabolism was disturbed in the PHC. 

Recent studies showed that intra-articular injections of certain specific miRNA had therapeutic effects for OA treatment. Most of the studies have focused on the therapeutic effect of miRNA on articular cartilage. In the surgical or drug-induced OA animals, mainly including rat and mouse models, miRNAs were effective for OA therapy by regulating some specific signals associated with the cartilage degeneration and OA development [15,37,38,39,40]. A couple of other studies concentrated on the therapeutic effects on the injured ligament or medial menisci [41,42]. Nevertheless, OA involves the whole joint rather than the articular cartilage degradation alone and the affected tissues include synovium, subchondral bone, meniscus, and ligaments [43]. In our study, the intra-articular injection of miR-199a-5p significantly inhibited the degeneration of articular cartilage tissue. The Ihh signaling and downstream factors associated with the chondrocyte hypertrophy and matrix degradation were significantly changed, which is consistent with the results observed in the PHCs. We further found that intra-articular injection of miR-199a-5p agomir had a certain therapeutic effect on synovial inflammation and subchondral bone degeneration. The mechanical stress-induced OA symptoms could rapidly mimic the OA progress triggered by joint weight-bearing and aging. However, which type of tissue causing the onset of OA and driving the whole joint destruction is not known [26]. miRNAs perform fine regulation on target genes, making them suitable for the treatment of OA, a complex disease involving multiple tissue degeneration [44]. Further study of the function and molecular mechanisms of miR-199a-5p on synovial tissue and subchondral bone are required. 

As a type of antisense oligonucleotides, miRNAs are short and the modified miRNAs are steady in vitro and in vivo. The time and tissue-specific characterization of miRNAs makes them useful for diagnosis and treatment in the clinic [45]. The expression of miR-199a-5p was decreased in the serum of the late-stage OA patients compared to the early-stage OA patients, which was consistent with our study that the expression of miR-199a-5p was downregulated in the damaged cartilage compared to undamaged cartilage in OA patients, and in OA PHCs compared to controls [16]. In the current study, intra-articular injection of miR-199-5p agomir could prevent cartilage damage and alleviate OA progression in early-stage OA rats. We did not study how the injected miR-199a-5p agomir affected colla2a1 and other extracellular matrix proteins with and without IHH-related effects, which is a limitation of the in vivo study. Nonetheless, in the present study, we regard a joint as a whole organ and investigate the pathological improvement of OA involving articular cartilage, synovial tissue, and subchondral bone. Our study demonstrates the potential clinical application of miR-199a-5p in the treatment of OA patients. 

In conclusion, this study showed that IHH and miR-199a-5p were related to damaged cartilage and chondrocytes. Further study revealed that miR-199a-5p could directly regulate IHH expression and reduce chondrocyte hypertrophy and matrix degradation via the IHH signal pathway in PHCs. The intra-articular injection of synthetic miR-199a-5p could attenuate articular cartilage destruction, subchondral bone degradation, and synovial inflammation, and inhibit the Ihh signal pathway in OA rats. This study might help in understanding the functional role of miR-199a-5p in the pathophysiology and molecular mechanisms of OA, thus indicating a potential novel therapeutic strategy for OA patients. 

## Data Availability

Data is contained within the article.

## Supplementary Materials

The following supporting information can be downloaded at: https://www.mdpi.com/article/10.3390/jcm12041313/s1, Table S1: Information on patients; Table S2: Primer information for qPCR and ad-IHH PCR; Table S3: Sequences of mimic/agomir NC and miR-199a-5p mimics/agomir; Table S4: Detailed information of antibodies. Table S5: The enrichment analysis of DEGs in Figure 1h.
