# Supplementary material for: miR-199a-5p Reduces Chondrocyte Hypertrophy and Attenuates Osteoarthritis Progression via the Indian Hedgehog Signal Pathway
Source: J Clin Med. 2023 Feb 7;12(4):1313. doi: 10.3390/jcm12041313 (PMC9959662; doi:10.3390/jcm12041313)
Supplement: Supplementary file 1 [file jcm-12-01313-s001.zip › jcm-2193475-supplementary.pdf]

Table S1: Information of patients.

Patient[a]	Gender	Age(years)	Height(kg)	Weight(cm)
1	F	70	55	160
2	F	63	70	160
3	M	71	59	156
4	F	73	65	168
5	F	68	65	165
6	M	71	62	167
7	F	71	75	156
8	F	59	69	163
9	M	66	60	172
10	F	63	55	160
11	F	68	55	161
12	F	72	75	163
13	F	70	76	160
14	M	67	65	158
15	M	58	82	170

a The articular cartilage of patient 1-3 was used for IHC, patient 4-6 was used for primary chondroctye culture and qPCR,

patient 7-9 was used for FISH, and patient 10-15 was used for primary chondroctye culture and western blot experiments.

F: female, M: male.

Table S2: Primer information for qPCR and ad-IHH PCR.

Name	Primer sequence (from 5' to 3')
IHH	Forward: ATCATCTTCAAGGACGAGGAGA
	Reverse: GGGCCTTTGACTCGTAATACAC
MMP3	Forward: CTGGACTCCGACACTCTGGA
	Reverse: CAGGAAAGGTTCTGAAGTGACC
MMP13	Forward: AATATCTGAACTGGGTCTTCCAAAA
	Reverse: CAGACCTGGTTTCCTGAGAACAG
COL2A1	Forward: GTTGGGAGTAATGCAAGGACC
	Reverse: CAGCTTCACCATCATCACCAG
miR-140[a]	Forward: CAGTGGTTTTACCCTATGGTAG
miR-199a-5p[b]	Forward: CCCAGTGTTCAGACTACCTGTTC
ad-IHH	Forward: AGCTGTGACCGGCGCCTACTCTGGTACCCGCCACCATGTCTC CCGCCCG
	Reverse: CTTATCTAGAAGCTTAGGCTCGAGTTATTTGTCGTCATCATCC TTATAGTCCTTA

a,b The reverse primers for miRNAs were universal primers from a universal miRNA one-step RT kit and
the primer sequence was not specified

Table S3: Sequences of mimic/agomir NC and miR-199a-5p mimics/agomir.

Name	Primer sequence (from 5' to 3')
mimic/agomir NC	Sense: UUCUCCGAACGUGUCACGUTT
	Antisese: ACGUGACACGUUCGGAGAATT
mimic/agomir miR-199a-5p	Sense: CCCAGUGUUCAGACUACCUGUUC
	Antisense: ACAGGUAGUCUGAACACUGGGUU

Table S4: Detail information of antibodies

Name	Company	Origin	Dilution
IHH	Abcam	MA, USA	WB 1:1000
			IHC 1: 200
GLI1	Abways	Shanghai, China	WB 1: 400
			IHC 1: 200
RUNX2	CST	MA, USA	WB 1: 1000
			IHC 1: 200
MMP3	Abcam	MA, USA	WB 1: 1000
MMP13	Bioss	Beijing, Chian	WB 1: 500
			IHC 1: 50
COL10A1	Abways	Shanghai, China	WB 1: 400
ADAMTS5	Bioss	Beijing, Chian	WB 1: 500
COL2A1	Boster	Wuhan, China	WB 1: 200
			IHC 1: 50
ACAN	Abways	Shanghai, China	WB 1: 400
			IHC 1: 200
GAPDH	Abways	Shanghai, China	WB 1: 5000

Table S5 The enrichment analysis of DEGs in Figure 1h.

Description	GeneRatio	pvalue	p.adjust	geneID
bone morphogenesis	31/1105	7.48E-12	6.95E-09	Igf1/Mbl1/Pthlh/Fgfr2/Ghr/Comp/Col2a1/Dlx5/Shox2/Alpl/Col10a1/Has2/Acp5/Serpinh1/Col1a1/Mmp16/Mmp14/Ihh/Fgfr3/Twist1/Smpd3/Mmp13/Ifitm5/Matn1/Col27a1/Sfrp2/Pax1/Vit/Osr2/Col6a2/Foxc1
bone development	44/1105	4.94E-11	3.44E-08	Gja1/Igf1/Mbl1/Pthlh/Sparc/Lox/Fgfr2/Ghr/Comp/Col2a1/Dlx5/Phex/Shox2/Alpl/Col10a1/Has2/Dbh/Acp5/Serpinh1/Col1a1/Ghrl/Smad5/Mmp16/Mmp14/Fbn1/Ihh/Fgfr3/Twist1/Smpd3/Tnfsf11/Mmp13/Ifitm5/Matn1/Col27a1/Dchs1/Fat4/Sfrp2/Pax1/Vit/Gpr68/Osr2/Col6a2/Foxc1/Srf
endochondral bone morphogenesis	21/1105	4.48E-09	1.04E-06	Mbl1/Pthlh/Ghr/Comp/Col2a1/Dlx5/Shox2/Alpl/Col10a1/Serpinh1/Col1a1/Mmp16/Mmp14/Ihh/Smpd3/Mmp13/Matn1/Col27a1/Vit/Col6a2/Foxc1
bone remodeling	15/1105	2.50E-03	2.72E-02	Egfr/Gja1/Tph1/Acp5/Pth1r/Ptprv/Tfrc/Ctnnb1/Ihh/Fgfr3/Inpp4b/Tnfsf11/Cthrc1/Lgr4/Fcgr3a
cartilage development	39/1105	8.41E-10	3.61E-07	Mbl1/Pthlh/Atp7a/Ghr/Comp/Col2a1/Shox2/Snai2/Col11a1/Bmp3/Col10a1/Nfib/Serpinh1/Col1a1/Tgfbr1/Pth1r/Acan/Smad5/Lum/Bmp1/Ctnnb1/Ihh/Fgfr3/Smpd3/Mmp13/Loxl2/Sox6/Col11a2/Adamts12/Matn1/Col27a1/Sfrp2/Vit/Osr2/Snx19/Adamts7/Arid5a/Col6a2/Srf
cartilage development involved in endochondral bone morphogenesis	14/1105	1.55E-06	1.46E-04	Mbl1/Ghr/Comp/Col2a1/Shox2/Serpinh1/Col1a1/Ihh/Smpd3/Mmp13/Matn1/Col27a1/Vit/Col6a2
chondrocyte differentiation	28/1105	1.19E-09	4.75E-07	Mbl1/Pthlh/Comp/Col2a1/Shox2/Snai2/Col11a1/Nfib/Serpinh1/Tgfbr1/Pth1r/Acan/Ctnnb1/Ihh/Smpd3/Loxl2/Sox6/Col11a2/Adamts12/Matn1/Col27a1/Sfrp2/Vit/Osr2/Snx19/Adamts7/Arid5a/Col6a2
chondrocyte	8/1105	1.55E-04	3.81E-03	Mbl1/Serpinh1/Ihh/Smpd3/Matn1/Col27a1/Vit/Col6a2

differentiation involved in endochondral bone morphogenesis				
chondrocyte proliferation	5/1105	3.81E-03	3.67E-02	Comp/Mmp16/Mmp14/Ihh/Ddr2
